# The Combined Impact of CLIR Post-Analytical Tools and Second Tier Testing on the Performance of Newborn Screening for Disorders of Propionate, Methionine, and Cobalamin Metabolism

**DOI:** 10.3390/ijns6020033

**Published:** 2020-04-10

**Authors:** Dimitar K. Gavrilov, Amy L. Piazza, Gisele Pino, Coleman Turgeon, Dietrich Matern, Devin Oglesbee, Kimiyo Raymond, Silvia Tortorelli, Piero Rinaldo

**Affiliations:** Biochemical Genetics Laboratory, Department of Laboratory Medicine and Pathology, Mayo Clinic, Rochester, MN 55905, USA; Piazza.Amy@mayo.edu (A.L.P.); Pino.gisele@mayo.edu (G.P.); Turgeon.Coleman@mayo.edu (C.T.); Matern@mayo.edu (D.M.); Oglesbee.Devin@mayo.edu (D.O.); Raymond.Kimiyo@mayo.edu (K.R.); Tortorelli.Silvia@mayo.edu (S.T.); Rinaldo@mayo.edu (P.R.)

**Keywords:** Collaborative Laboratory Integrated Reports (CLIR), false positive rate, newborn screening (NBS), second tier test (2TT)

## Abstract

The expansion of the recommend uniform screening panel to include more than 50 primary and secondary target conditions has resulted in a substantial increase of false positive results. As an alternative to subjective manipulation of cutoff values and overutilization of molecular testing, here we describe the performance outcome of an algorithm for disorders of methionine, cobalamin, and propionate metabolism that includes: (1) first tier screening inclusive of the broadest available spectrum of markers measured by tandem mass spectrometry; (2) integration of all results into a score of likelihood of disease for each target condition calculated by post-analytical interpretive tools created byCollaborative Laboratory Integrated Reports (CLIR), a multivariate pattern recognition software; and (3) further evaluation of abnormal scores by a second tier test measuring homocysteine, methylmalonic acid, and methylcitric acid. This approach can consistently reduce false positive rates to a <0.01% level, which is the threshold of precision newborn screening. We postulate that broader adoption of this algorithm could lead to substantial savings in health care expenditures. More importantly, it could prevent the stress and anxiety experienced by many families when faced with an abnormal newborn screening result that is later resolved as a false positive outcome.

## 1. Introduction

Over the last two decades, the introduction of tandem mass spectrometry (MS/MS) for the simultaneous analysis of acylcarnitine and amino acid species in dried blood spots (DBS) [1] has greatly expanded the number of target conditions [2]. Currently, the US Secretary of Health and Human Services recommends screening for a panel of 34 conditions [3], more than half of them can be detected only by MS/MS methodology. Traditionally, the interpretation of an abnormal MS/MS result for a given analyte has been based on the comparison to cutoff values (high, low, or both) chosen by one of alternative methodologies (percentiles, multiple standard deviations added to the mean value, threshold of disease ranges) [4]. Once a result has been flagged as potentially abnormal, a more in depth evaluation is usually performed through the calculation of ratios to determine its significance according to established protocols, and eventually it triggers referral to follow up. Although this approach has been and continues to be used extensively, it soon became obvious that false positive test results [5] and also the risk of false negative events [6,7] were too common to be resolved without improved strategies. An obvious requirement is that any sequential intervention should not require additional patient contact, and therefore the only available choice has to be more testing of the same blood spot used by the first tier method. Indeed the development of second tier tests (2TT) became a major focus of our laboratory shortly after becoming a provider of newborn screening services in 2004. It was evident that the two most common causes of false positives by MS/MS were tyrosine and propionylcarnitine and they were prioritized accordingly [8,9]. Further method development led to the incorporation of succinylacetone into the first tier analysis [10].

There are many disorders of propionate, cobalamin, and methionine metabolism and their differential diagnosis at the biochemical level is complex (Table 1). Enzymatic deficiencies of propionyl-CoA carboxylase and methylmalonyl-CoA mutase are associated, respectively, with Propionic acidemia and several complementation groups of Methylmalonic acidemia that have in common an elevation of propionylcarnitine (C3) in DBS. C3 is also elevated in inherited and acquired conditions leading to deficiencies of vitamin B12 (cobalamin, Cbl), often related to maternal conditions (e.g., pernicious anemia, short gut syndrome) or dietary practices (e.g., vegetarian diet, macrobiotic diet). Inherited conditions of Cbl absorption and transport are caused by mutations in the genes encoding Cbl binding factors, trans-membrane transporters, and receptors [11]. In addition to Homocystinuria, isolated elevation of methionine (Met) is the biochemical marker of several inherited defects of the conversion of methionine to homocysteine. High Met can also be secondary to dietary interventions (total parenteral nutrition (TPN), catabolic state, etc.), or reflect significant liver disease [12]. Finally, low Met concentration is the first tier indicator of three disorders of remethylation [13], and also the trigger of the same 2TT intervention [14]. Overall, at least 20 conditions leading to abnormal concentration of C3 and/or Met in various combinations have been described (Table 1), arguably representing the most complex differential diagnosis to be confronted in the post-analytical interpretation of newborn screening of inborn errors of metabolism by MS/MS. Although treatment strategies for these disorders differ according to the specific group or particular entity, some general principles can be applied: prompt hospitalization, low protein diet, carnitine supplementation, and additional pharmacological interventions. On the other hand, precautionary implementation of these aggressive measures in cases later resolved as false positive events can be highly traumatic for patients and their families [15], and also quite expensive.

In addition to pioneering 2TTs for markers measured by MS/MS, our laboratory has also been leading the development and implementation of the collaborative project Region 4 Stork (R4S) in pursuit of performance improvement [16]. The initial phase of R4S focused on clinical validation of high and low target ranges for cutoff values [5]. Once it became apparent that such an approach was not widely adopted, the focus shifted to multivariate pattern recognition software that generates post-analytical interpretive tools. The novelty of these tools rested on replacing analyte cutoff values with the degree of overlap, calculated by comparison of data converted to Multiples of the Median (MoM), between reference and condition-specific disease ranges; and also on the provision of an interpretation based upon an objective measure of likelihood of disease [17,18]. By the time R4S was sunset in September 2018, it had been deployed in 69 countries by 269 laboratories, more than 1200 users were active and data were collected from 20,938 individual cases affected with one of 94 inherited and acquired conditions. The cumulative (2011–2018) utilization of the tools exceeded 411 million calculated scores. R4S has been replaced by Collaborative Laboratory Integrated Reports (CLIR; https://clir.mayo.edu), a second generation software with the notable addition of covariate-adjusted reference and disease ranges applicable to DBS collected up to one year age (R4S was validated only for use in the first 10 days of life), paving the way to the concept of precision newborn screening [19], which is the achievement of a <0.01% false positive rate per test without additional patient contact and before molecular testing. In 2019 alone, CLIR tools were utilized 238 million times worldwide, more than 650,000 times per day.

## 2. Materials and Methods

### 2.1. Analytical Methods

The methods for first tier testing by FIA-MS/MS of butyl-ester amino acids and acylcarnitines [10] and for the measurement by LC-MS/MS of methylmalonic acid (MMA), 2-methylcitric acid (MCA), total homocysteine (tHcy), and succinic acid (SUCC) in dried blood spots have been reported previously [14,20]. They all are laboratory developed tests.

### 2.2. Reference Ranges

As of January 31, 2020, CLIR cumulative reference ranges for C3 and methionine were based on 3,072,038 and 2,578,567 data points, respectively, from 77 and 81 programs. The ranges of age at collection and birth weight are 1–8760 h (median 26 h) and 250–9802 grams (median 3375 grams), respectively. Data submission is guided by a mandatory validation tool that automatically removes for each marker values above the cumulative 99% percentile of accepted data +20% and below the midpoint between the cumulative 1% percentile and zero. For low concentration markers, values <0.01 nmol/mL are also rejected. Further filtering that is left at the discretion of each location removes values either >5 Multiple of the Median (MoM) or <0.2 MoM. For the 2TT, after an initial validation with 200 control DBS [20], our established process in support of constantly evolving validation is data mining of clinical data on a frequent schedule. All profiles reported with a normal interpretation are compiled and uploaded to CLIR, growing to a current count of 5091 cases. Application of the Data Validation Tool is performed as described above.

### 2.3. Covariate Adjustments

Each marker measured by first-tier MS/MS is adjusted for age, birth weight, and location using a statistical normalization technique according to a regression-based method [21]. Markers of the 2TT are adjusted only for age as the vast majority of the samples were external and submitted without a value for birth weight.

### 2.4. Disease Ranges

As of January 31, 2020, 816 positive cases with disorders of propionate, cobalamin, and methionine metabolism are included in the CLIR database (Table 1). There are also 124 C3 false positive cases confirmed by sequencing [22], and 2816 cases with abnormal amino acid profiles secondary to TPN. Notably, not all cases previously submitted to R4S have been migrated to CLIR either for lack of permission, location drop out, or practical difficulties to link data to covariates, which is a mandatory requirement. The count of positive cases by the 2TT is 253, divided in six descriptive groups (see results).

### 2.5. CLIR Tools

**Figure 1:**
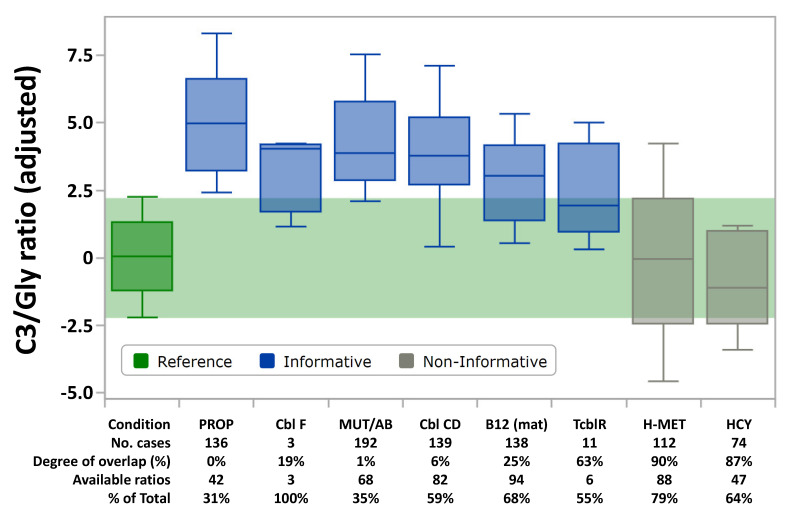
CLIR productivity tool “Plot by Marker” for C3/glycine ratio. Box and whisker plots display the 99%, 90%, 50%, 10%, and 1% percentiles, respectively. The horizontal green band overlays the reference percentiles (dark green, *n* = 1,368,748) with the individual disease ranges. Ranges are shown as Multiple of the Median after simultaneous adjustment for age (hours), birth weight (grams), and location (*n* = 50), expressed as Z-scores. Condition codes are the same as shown in Table 1.

Taking into consideration the merging of diseases with overlapping phenotypes, nine first tier Post-Analytical Tools have been released for the conditions listed in Table 1. These tools evaluate all markers and calculated ratios simultaneously that are selected as informative [17]. A marker is deemed to be “informative” when the degree of overlap between the high (99%ile) or low (1%ile) reference percentiles and the condition-specific disease range is less than 50%, meaning that the median of the disease range, calculated as MoM, is outside of the reference percentiles. In addition to C3 and Met, the count of ratios per tool varies between 17 and 21 (see Figure S1). The variability in ratio selection is a case by case compromise between low degree of overlap and high count of data points: for example, the ratio C3/glycine shows a complete separation (zero degree of overlap) between disease and reference range, but is available in only 42 (31%) of 136 cases with Propionic acidemia (Figure 1). Considering Propionic acidemia was originally described as Ketotic Hyperglycinemia, it is ironic that exclusion of this ratio from the default shared tool is a necessity, but it could be added to any site-specific tool at the discretion of the designated Tool Editor for that location.

**Figure 2:**
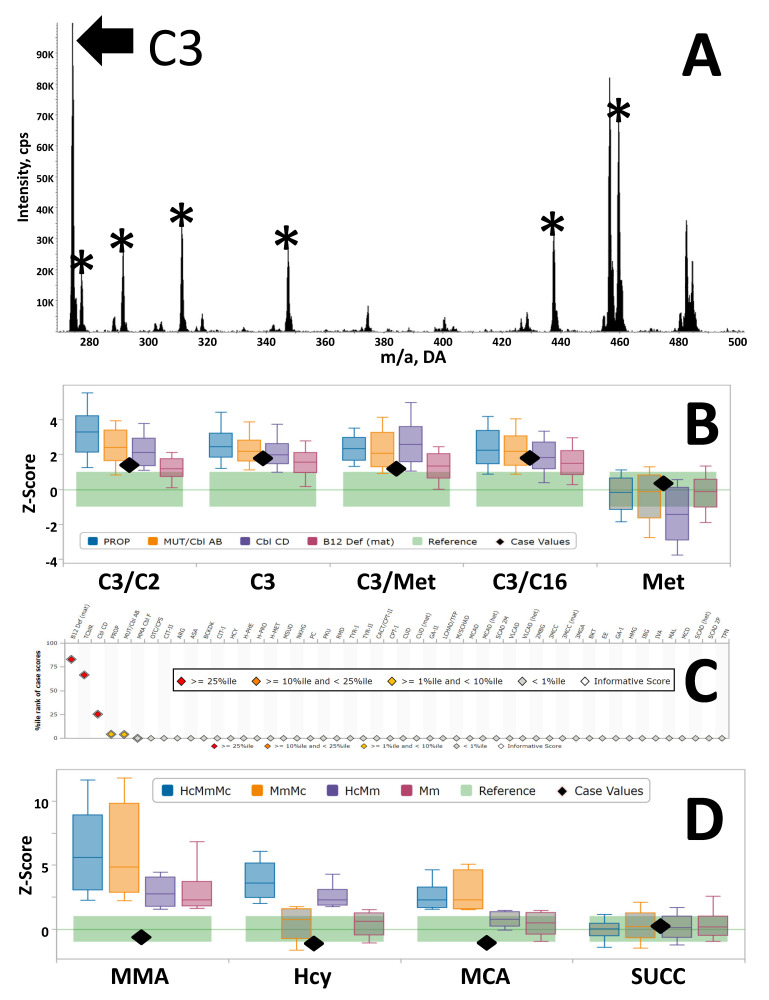
Panel (**A**) Acylcarnitine profile of a case with elevated C3 as first tier result, p85 profile of butyl-ester species. *, Internal standards [18]. Panel (**B**) Acylcarnitine profile of a case with elevated C3 as first tier result, p85 profile of butyl-ester species. *, Internal standards [18]. Panel (**C**) CLIR post-analytical tool “All Conditions Tool”. Color coding of scores is according to the embedded legend. Y-axis is the %ile rank of case scores (likelihood of disease), conditions are sorted highest to lowest from the left. Panel (**D**) CLIR productivity tool “Plot by Multiple Conditions” for the four markers measured by the second tier test (2TT). Black diamonds arranged as in Panel B. Concentrations were as follows: MMA 0.3 nmol/mL (test catalog reporting cutoff <5); tHcy 0.9 nmol/mL (<15); MCA 0.1 nmol/mL (<1). Abbreviations of conditions are as follows: Hc, isolated elevation of tHcy; HcMmMc, combined elevated concentrations of tHcy, MMA, and MCA; MmMc, combined elevated concentrations of MMA and MCA; HcMm, combined elevated concentrations of tHcy and MMA; Mc, isolated elevation of MCA; Mm, isolated elevation of MMA. Ranges are shown as Multiple of the Median after simultaneous adjustment for age (hours), birth weight (grams), and location.

Any commercial MS/MS software can be programmed to generate data output in comma-separated file (.csv) format, already inclusive of LOINC [23]. These files can be analyzed using the Tool Runner functionality [18], the calculation of a score for all released tools is completed in a few seconds for a standard load of 96 samples per plate. When a profile is flagged as potentially informative for one or more conditions (see panel C of Figure 2), further evaluation with multiple paired Dual Scatter Plots can be performed rapidly by a trained user to reach a decision if performing the 2TT is indicated (for example, the Dual Scatter Plots between Methylmalonic acidemia Cbl CD and maternal vitamin B12 deficiency, see Figure S2). If the 2TT is not reflexed, a case is reported with a standard interpretation that no abnormalities were detected, without mentioning the potentially abnormal result(s) detected by first tier screening. A normal 2TT result overrides the initial observation, and a normal report is released as mentioned above. A practical example of the clinical utility of DSP tools has been reported recently [24].

## 3. Results

### 3.1. Outcome of Second Tier Test

In our practice, the 2TT is required in approximately 1% of all newborns tested by the first tier assay. For example, in 2012–2013, our laboratory performed the primary screening by MS/MS for 142,517 newborns born in Minnesota. Because of a legislative mandate by Minnesota Statute 144.125 following a ruling by the State Supreme Court, all data and interpretations obtained at Mayo Clinic between 2004 and 2013 had to be deleted permanently at any level of either electronic or printed storage. The only information still available is the number of 2TTs performed (1618, 1.1% of the births) and the count of true positive cases detected over that period (4, 0.25% of the 2TTs). All cases had a diagnosis related to elevated C3, none was linked to Met. During the same period, we performed an additional 1416 tests for patients less than 1 year of age from outside Minnesota, but there is no information available regarding what proportion of the total number of cases they represent, the number of abnormal results was 78 (5.5% of the 2TTs presumably triggered by first tier testing). Between 2014 and 2019, we performed 4444 additional 2TTs for clinical use in the same age range, 175 (3.9%) were abnormal. In summary, our experience suggests that the 2TT is indicated in approximately 1% of births as follow up for abnormal C3 and/or Met results by first tier testing. When the test is performed, our multi-year experience shows that approximately 5% of specimens yield an abnormal result. Even in the rather unlikely event that all of them are later resolved as false positive cases, there is still a strongly beneficial outcome by preventing ~95% of potential false positive outcomes.

### 3.2. Example of Clinical Utility

An example of the clinical utility of the 2TT for elevated C3 is illustrated in Figure 2. Panel A shows the MS/MS acylcarnitine profile of a patient born at term (birth weight 3050 g, gestational age 38 weeks), sample collected at 59 h of age. The C3 concentration was 6.66 nmol/mL (99% percentile of unadjusted reference range is 3.59 nmol/mL), a value that is also near the 90% percentile of the range of cutoffs adopted by 176 programs [5]. In other words, this is a finding that likely would trigger a referral by a vast majority of laboratories, especially when considering that commonly used ratios were also elevated (C3/C2: 0.30, 99%ile of reference range is 0.19; C3/C16: 2.19, 99%ile 1.86; C3/Met: 0.31, 99%ile 0.23) (Figure 2B, shown as adjusted values). Methionine was normal (22.98 nmol/mL, 1–99%ile range is 10–40). This profile resulted in an informative score for all C3-related interpretative tools (Figure 2C).Yet, the 2TT showed a completely negative profile (Figure 2D, measured values included in the figure legend), which was the basis for overriding with confidence the first tier result leading to a negative report void of any mention of the initial finding.

### 3.3. Distribution of Abnormal Cases

**Figure 3:**
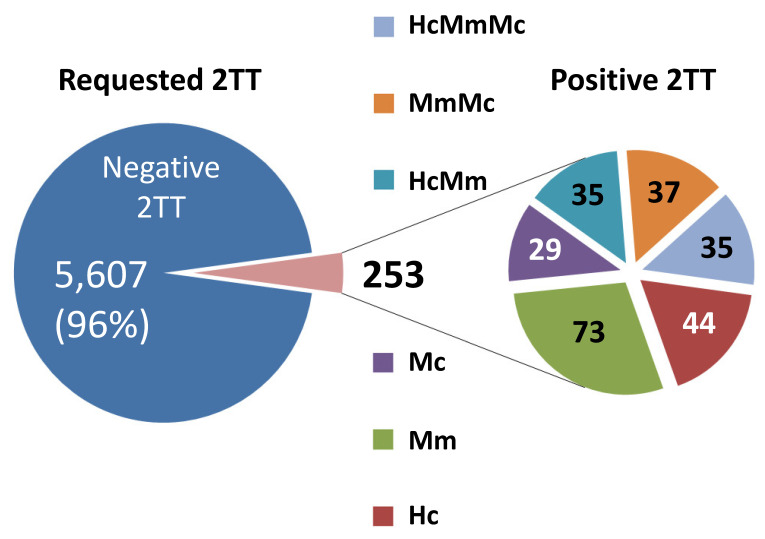
Distribution of cases with an abnormal 2TT. Hc, isolated elevation of tHcy; Mc, isolated elevation of MCA; other abbreviations are as in the legend of Figure 2.

The classification of conditions after an abnormal 2TT is hampered by the limited clinical feedback returned once requested and also by the intrinsic uncertainty of the exact nature of the result (for example, a markedly elevated homocysteine could be secondary either to Homocystinuria or to a remethylation disorder). Samples are received in most cases as a single 3 mm punch preventing the option of requesting permission to perform an offline verification of the first tier testing on a backup punch. On the other hand, the foundation of the clinical utility of CLIR is the concept of constantly evolving clinical validation by addition to the database of newly discovered cases. As a compromise, we have classified cases informative by the 2TTs according to a nomenclature that simply reflect the isolated or combined findings with codes as listed in Figure 2. Figure 3 shows the distribution of abnormal cases sorted as described above for the period 2012–2019. An isolated elevation of MCA was considered to indicate Propionic acidemia, and these cases accounted for 29 (11%) of the 253 abnormal results. An isolated elevation of tHcy in 44 (17%) of the abnormal results was taken as indication of either Homocystinuria or a remethylation disorder. An elevation of all three markers was found in 35 cases (14%) likely affected with Cbl CD, but other less common cobalamin disorders and also severe maternal vitamin B12 cannot be ruled out. The final 145 cases (57%), showing either an isolated elevation of MMA or combined with either tHcy or MCA, are less predictable in terms of the actual underlying condition, suggesting a need to improve our efforts to obtain clinical outcome feedback in a much greater proportion of cases.

### 3.4. Actual and Potential Cost Savings

Data on the financial impact of false positive newborn screening (NBS) results are anecdotal. The publication of a detailed analysis of the cost savings achieved in Minnesota (2004–2013) regrettably has been vetoed by the IRB committee of the Minnesota Department of Health because of concerns it could amount to a violation of the 2012 Minnesota statute. Nevertheless, 1.1% of the population tested required the 2TT, only 4 (0.003% of the tested population) were resolved as true positives. Even assuming 16 hypothetical false positives (four times the number of confirmed case, a proportion chosen arbitrarily) among the 1614 cases with abnormal results, the false positive rate would remain <0.01%, the proposed threshold of precision newborn screening. In term of cost savings due to prevented referral and follow up, a rough estimate can be made when considering the ACT sheets and algorithms that were developed by the American College of Medical Genetics and Genomics (ACMG) to facilitate timely and proper clinical and laboratory follow-up of abnormal NBS results [25]. Based on data from FAIR Health, Inc., the eight first-line routine and specialty laboratory tests recommended by the ACT sheet for C3 could result in a charge of up to $660 for phlebotomy and laboratory services. To these charges fees must be added for at least one physician visit, keeping in mind that for an abnormal C3 referral, the patient is likely to be directed to a metabolic center and/or an emergency room, especially when screening results are acted upon outside of regular business hours. Assuming a 2:1 ratio of babies still seen in a primary care vs. emergency setting, an additional consult with a Medical Geneticist, the professional and facility charges could add up to another $640. Overall, the total average charge of the first instance of follow-up for an abnormal C3 result could be estimated at approximately $1500. Similar charges could be applied for persistently elevated Met after the repeat analysis, if 2nd tier testing for tHcy is not done at this time.

### 3.5. The Price of Being Wrong

**Figure 4:**
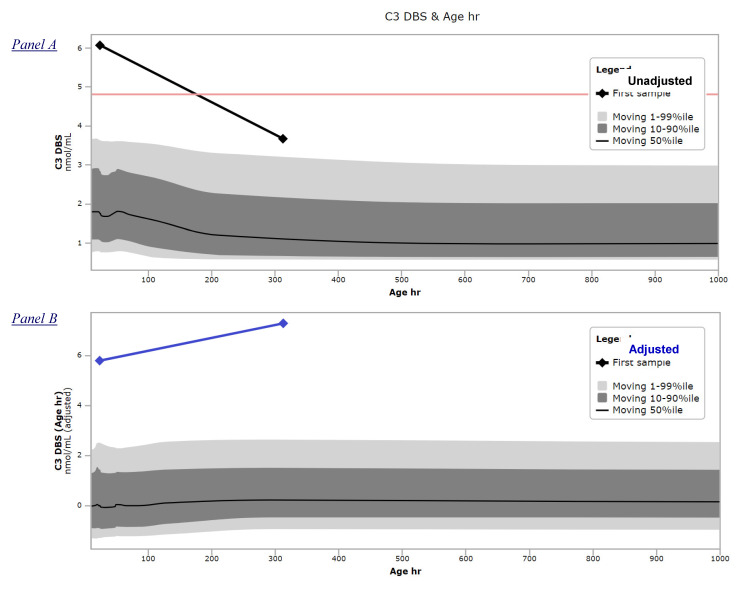
CLIR productivity tool “Marker vs. Covariate plot”. Panel (**A**) Unadjusted moving percentiles of C3 actual values in the age range 10–1000 h, linear scale. Black diamonds, connected by a line, represents from left to right the C3 values of the initial (6.07 nmol/mL) and repeat (3.67 nmol/mL) samples, respectively. Red line corresponds to the cutoff value of the testing laboratory in use at the time. Panel (**B**) Adjusted moving percentiles of C3 in the age range 10–1000 h. Blue diamonds, connected by a line, represent from left to right the C3 values after adjustment for age of the initial and repeat samples, respectively.

Cost estimates like those above may be seen as disproportionate because “all it takes” is the collection of a repeat sample. C3, however, is in a uniquely dangerous situation because there is a substantial decrease of the reference range between the usual age of collection (in the US 24 h) and 6 weeks of age. In other words, the pervasively used practice of a single cutoff values for all ages is likely to interpret a repeat value as normal even if well above the actual 99%ile for a later age. The serious consequences of a situation of this kind, summarized in Figure 4, have been highlighted in a highly publicized newspaper article [26]. Briefly, an abnormal result by first tier testing (C3 6.07 nmol/mL) was overruled by the finding of a lower result (3.67 nmol/mL) in the repeat sample collected at 13 days of age. Both values were interpreted using the same cutoff value shown in Figure 4A as a red line. However, Figure 4B shows that after adjustment for age in CLIR, the repeat sample actually corresponded to a higher adjusted value in comparison to the initial one. No complete data of this case are publicly available to run the profile against the CLIR tools, but the degree of abnormality of the adjusted C3 concentration makes it highly probable that at least one informative score could have been obtained to suggest performing the 2TT (routinely available at the time), and if that was done, likely could have prevented this tragic false negative event.

## 4. Discussion

After 15 years of clinical utilization, there is strong evidence that the biochemical 2TTs for disorders of propionate, methionine, and cobalamin metabolism have contributed to performance improvement of newborn screening by MS/MS, a reality validated by its replication worldwide with a combination of modifications and enhancements [27,28,29,30,31,32]. Like in the case of succinylacetone [10], it is now possible to analyze tHcy as part of the first tier screening when warranted by a high local incidence of Homocystinuria [33]. Evidence of similar clinical utility has been observed for additional first tier MS/MS markers with poor specificity [34,35], and other groups of conditions as well [36,37,38], so the trend of developing new tests is likely to continue, starting with one new application also reported in this special issue [39]. Furthermore, to specifically resolve the intrinsic ambiguity observed in this cohort of isolated elevation of tHcy, we are in the process of adding methionine, cysteine, and cystathionine to the 2TT panel.

2TTs should not be expected to be a complete substitute for molecular testing, rather they are complementary components of the newborn screening system. Yet, higher maturity and dependability should be achieved in the interpretation of variants beyond the too frequent classification as “uncertain significance” [40]. To that extent, perhaps greater consideration and evidentiary weight could be given to biochemical evidence so it could become an accelerator toward the delivery of greater benefits for large number of patients. Significant financial savings, achievable by this approach and illustrated by placing individual savings as calculated above in the context of many instances occurring on a daily basis nationwide, could prevent significant health care expenditures without even accounting for the financial and emotional burden on families that are forced to cope with the prospect of their newborn being affected with a serious and chronic medical condition.

The implementation of a full menu of biochemical 2TTs by every program, perhaps with the exception of those with a number of births >150,000 per year (national programs with a single testing laboratory and a few US states) is hindered by capacity constraints and needed resources. However, a regionalization effort with individual programs, each becoming the provider of one or more 2TTs for a given geographical area, offered as an in-kind “trade” rather than a service, could lead to full coverage and efficient utilization. Alternatively, reliance on a referral laboratory either academic or commercial is a valid alternative, a model that has being gaining momentum especially among US programs in the process of adding lysosomal disorders to their mandated panels.

## Figures and Tables

**Table 1:** Markers of inherited and acquired disorders of propionate, cobalamin, and methionine metabolism, count of cases, and Collaborative Laboratory Integrated Reports (CLIR), tools.

Disorder	Compl. Group	OMIM #	Gene	1st Tier Markers		CLIR		2nd Tier Markers		
				C3	Met	* No. Cases	MS/MS Tool	Hcy	MMA	MCA
**Propionic acidemia**	**n/a**	**606054**	PCA, PCB	High	N	136	PROP	N	N	High
Isolated Methylmalonic acidemia	mut^0^ mut^-^	251000	MCM			192	MUT/Cbl AB	N	High	High
	Cbl A	251100	MMAA							
	Cbl B	251100	MMAB							
Methylmalonic acidemia and Homocystinuria	Cbl C	277400	MMACHC		Low	139	Cbl CD	High	High	N to High
	Cbl D	277410	MMACHC							
	Cbl F	277380	LMBRD1			3	Cbl F			
	Cbl J	614857	ABCD4			-	-			
	Cbl X	309541	HCFC1			-	-			
Intrinsic factor deficiency	n/a	261000	GIF		N	-	-	High	High	N to High
Megaloblastic anemia-1		261100	CUBN, AMN			-	-			
Transcobalamin II deficiency		275350	TCN2			-	-			
Transcobalamin receptor defect		613646	CD320			11	TCblR			
Maternal Vitamin B12 deficiency		-	-		Low	138	B12 (mat)			
Homocystinuria (CBS deficiency)	n/a	236200	CBS	N	High	74	HCY	High	N	N
Homocystinuria and megaloblastic anemia	Cbl G	250940	MTR		Low	11	RMD			
	Cbl E	236270	MTRR							
MTHFR deficiency	n/a	236250	MTHFR							
Methionine adenosyltransferase def.		250850	MAT 1A		High	112	H-MET	N	N	N
Adenosine kinase deficiency		180960	ADK							
Glycine N-methyltransferase def.		606664	GNMT							
S-adenosylhomocysteine hydrolase def.		613752	AHCY							
FP C3		n/a	n/a	High	N	124	FP C3	N	N	N
TPN		n/a	n/a	N	High	2816	TPN	N	N	N

* Count of CLIR cases as of January 31, 2020. Abbreviations as follows: C3, propionylcarnitine; Cbl, cobalamin; CBS, cystathione β-synthase; CLIR, Collaborative Laboratory Integrated Reports (see text); FP, false positive; Hcy, total homocysteine; High, elevated concentration in dried blood spots in >50% of cases; Low, reduced concentration in dried blood spots in >50% of cases; Met, methionine; MCA, 2-methylcitric acid; MMA, methylmalonic acid; mut, mutase; MTHFR, (N)5,10-methylenetetrahydrofolate reductase; n/a, not applicable; N, normal concentration in dried blood spots; N to High, inconsistent elevation in <50% of cases; RMD, remethylation disorders, OMIM #—Online Mendelian Inheritance in Man symbol indicating a descriptive entry, usually phenotype.

## Supplementary Materials

The following are available online at https://www.mdpi.com/2409-515X/6/2/33/s1.
