# Supplementary material for: The Combined Impact of CLIR Post-Analytical Tools and Second Tier Testing on the Performance of Newborn Screening for Disorders of Propionate, Methionine, and Cobalamin Metabolism
Source: Int J Neonatal Screen. 2020 Apr 10;6(2):33. doi: 10.3390/ijns6020033 (PMC7423003; doi:10.3390/ijns6020033)

Figure S1

CLIR plot by condition showing the two markers and 20 ratios considered for the calculation of a score by the single condition tool for condition **Cbl CD (combined Methylmalonic acidemia and Homocystinuria)**. Reference (1-99%ile, green shade) and disease (blue box and whisker plots of 1%ile, 10%ile, median, 90%ile,and 99%ile of each marker) ranges are shown as Z-scores of values adjusted simultaneously for age at collection, birth weight, and location (N=78 for C3; count varies for other markers). Informative status is assigned when the median of a disease range is outside of the peripheral percentiles (see text). Degree of overlap is based on adjusted values. Abbreviations are as follows: C0. free carnitine; C2, acetylcarnitine; C3, propionylcarnitine; C4, butyrylcarnitine+Isobutyrylcarnitine; Isovalerylcarnitine+methylbutyrylcarnitine; C8 octanoylcarnitine; C14:1, tetradecenoylcarnitine; C16, palmitoylcarnitine; C16OH, hydroxypalmitoylcarnitine; C18:1, oleylcarnitine; C18, stearoylcarnitine; Cit, citrulline; Met, methionine; Phe, phenylalanine; Tyr, tyrosine; Val, valine; Xle, isoleucine+leucine

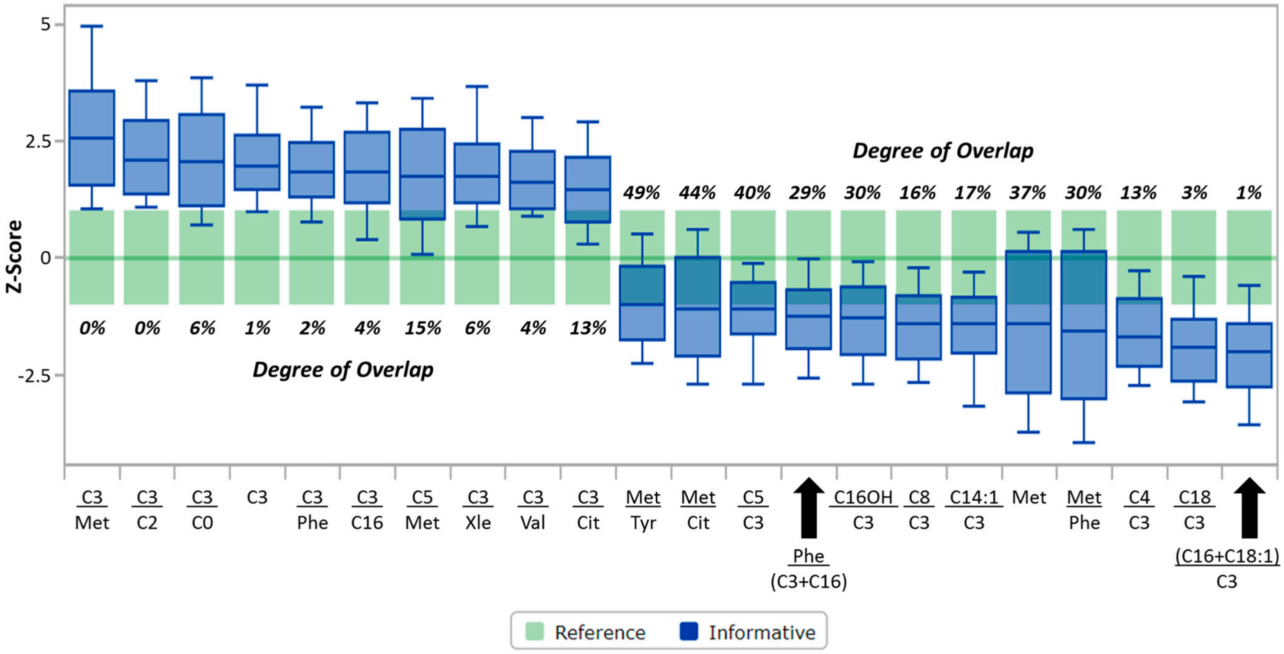

Figure S2

CLIR dual scatter plot applied to the differential diagnosis between Methylmalonic acidemia Cbl CD and maternal vitamin B12 deficiency. The plot is divided in four quadrants and a central square: Lower right: consistent with Cbl CD (light blue circles and squares; a square indicates a case from the primary location of the user); Upper right: Neither condition; Upper left: consistent with B12 def (mat) cases (purple circles and squares); Lower left: neither condition. The central square (magnified in the insert in the upper right corner) represents cases of both conditions that cannot be resolved by this tool as one condition or the other. In consideration of the strong similarity between the biochemical phenotypes, it

is noteworthy that 74% and 49%, respectively, of the two conditions can be separated on the sole basis of the first tier screening. For a description of the min-max score normalization, see references [17,18].

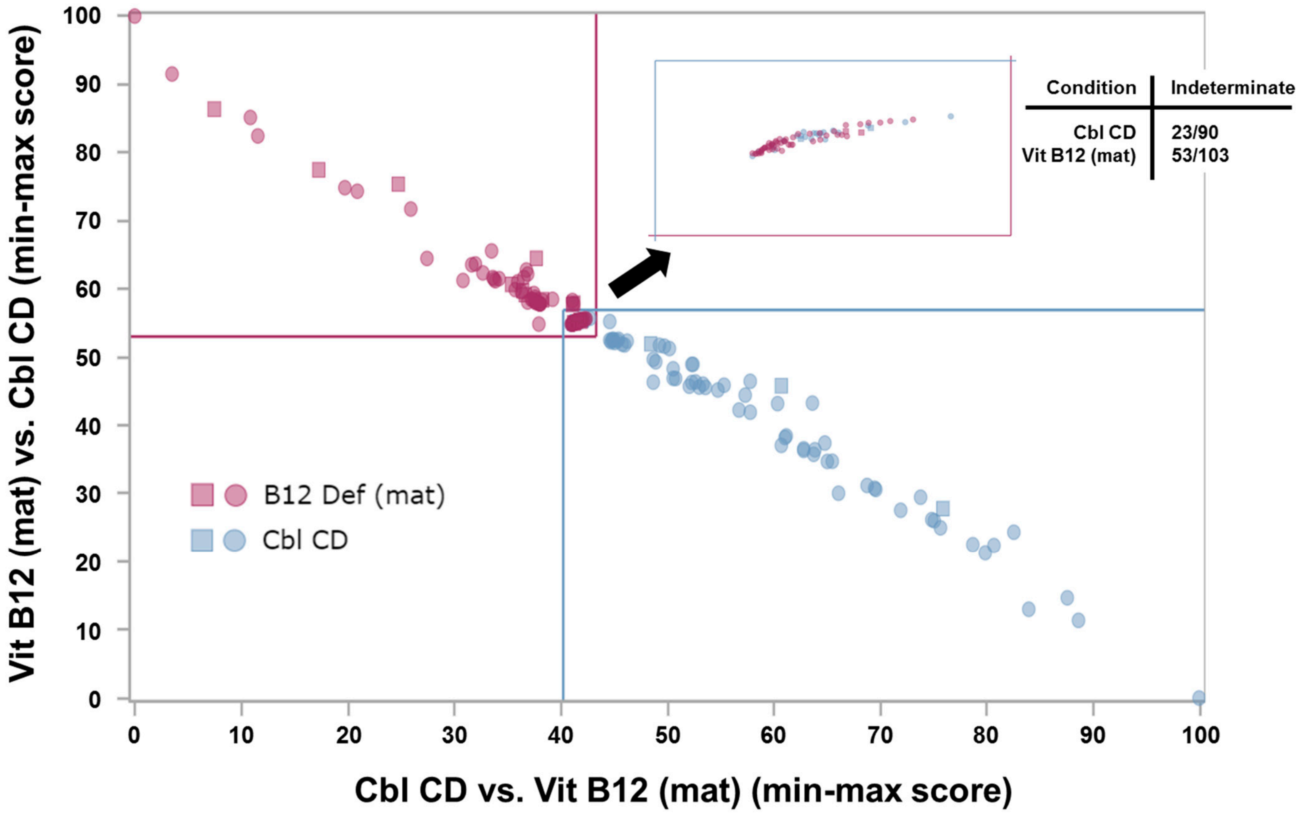

Supplement: Supplementary file 1 [file IJNS-06-00033-s001.pdf]
